# Effect of Regular Exercise on Inflammation Induced by Drug-resistant *Staphylococcus aureus* 3089 in ICR mice

**DOI:** 10.1038/srep16364

**Published:** 2015-11-06

**Authors:** Jong-Kook Lee, Tudor Luchian, Yoonkyung Park

**Affiliations:** 1 Research Center for Proteinaceous Materials (RCPM), Chosun University, Gwangju, Korea; 2 Department of Physics, Alexandru I. Cuza University, Iasi, Romania; 3 Department of Biotechnology & BK21-Plus Research Team for Bioactive Control Technology, Chosun University, Gwangju, Republic of Korea

## Abstract

Obesity is often associated with irregular dietary habits and reduced physical activity. Regular exercise induces a metabolic response that includes increased expression of various cytokines, signaling proteins and hormones, and reduced adipocyte size. In this study, mice performed a swimming exercise for 10 min/day, 5 days/week for 3 weeks. We then investigated the effect of this exercise regimen on inflammation induced by infection with drug-resistant *Staphylococcus aureus* strain 3089 (DRSA). In humans, DRSA causes dermatitis and pneumonitis. Similarly, DRSA induced inflammatory pneumonitis in both no-exercise (No-EX) and swim-trained (SW-EX) ICR mice. Regular exercise increased levels of the pro-inflammatory cytokines TNF-α and IL-1β and nitric oxide in both serum and whole lung tissue in SW-EX, as compared to No-EX control mice. Moreover, levels of the antimicrobial peptide cathelicidin were significantly increased in visceral adipose tissue and whole lung tissue in the SW-EX group, and this was accompanied by a reduction in the size of visceral adipocytes. In addition, levels of the inflammation marker peroxisome proliferator-activated receptor gamma coactivator-1 (PGC-1) were not increased in the lung tissue of SW-EX mice. These findings suggest that in these model mice, regular exercise strengthens immune system responses, potentially preventing or mitigating infectious disease.

In recent years, rapid improvements in industrial and informational technology have increased the quality of life for many^1^. On the other hand, there has been an increase in environmental pollution, and people tend to do less regular exercise and suffer greater amounts of stress^2^. These conditions have led to an increased incidence of obesity and greater exposure to bacterial and viral diseases^3^,^4^,^5^,^6^,^7^,^8^. Moreover, overuse of antibiotics has led to the development of drug-resistant microorganisms such as methicillin-resistant *staphylococcus aureus* (MRSA), vancomycin-resistant *enterococci* (VRE) and other multi-drug resistant (MDR) strains, which are the source of potentially life-threatening infections in the absence of a robust immune response^9^. This is particularly true in hospitals, where recent reports indicate that the drug resistance of various strains has increased the death rates from nosocomial infections^10^,^11^.

Obesity is associated with chronic low-grade inflammation, an increased risk of atherosclerosis, type 2 diabetes, neurodegeneration, tumor growth, reduced functional capacity and reduced longevity in humans^3^,^12^. Cytokines are signaling molecules secreted by various cell types, which control immune responses and are involved in the regulation of cell proliferation and differentiation and antitumor activity, among other functions^12^,^13^,^14^. A lack of physical activity has been associated with increased production of pro-inflammatory cytokines, adipokines (a subgroup of cytokines produced by adipose tissue), triglycerides and LDL-cholesterol, as well as overexpression of Toll-like receptors (TLRs), including TLR2 and TLR4^15^,^16^,^17^,^18^,^19^,^20^,^21^,^22^. Conversely, regular exercise reportedly increases production of various cytokines and myokines (a subgroup of cytokines produced in skeletal muscle) that strengthen the immune system, while stimulating increases in adipose tissue oxidation, insulin sensitivity, anti-inflammatory activity, anti-tumor defense, pancreatic function, and brown fat metabolism for muscle growth^23^,^24^,^25^,^26^.

The secretion of antimicrobial peptides (AMPs) such as cathelicidin and defensin by immune cells is one of the ways in which the body protects itself from disease, including antibiotic-resistant bacterial infections^27^. In particular, cathelicidin secreted by macrophages and polymorphonuclear leukocytes (PMNs) is well known to exert strong antimicrobial effects^27^. Given the beneficial effects of the exercise on immune system function summarized above, we hypothesized that a lack of exercise increases the potential for serious organ damage as well as increased morbidity and mortality among those who become ill. Consistent with that idea, regular exercise appears to increase immunity and reduce metabolic syndrome disease acitivity^28^,^29^,^30^. Furthermore, we suggest that regular exercise increases cathelicidin production by immune cells, which, in the event of an infection, would reduce the growth and destructive effects of the microorganism.

To test those ideas, we applied swim training to ICR mice and compared the responses to infection by drug-resistant *Staphylococcus aureus* strain 3089 (DRSA) in no-exercise (No-EX) and swim-trained (SW-EX) groups. Susceptibility to DRSA and manifestation of its symptoms (e.g., dermatitis and pulmonitis) reflect the host’s ability to mount an appropriate immune response^31^. We anticipated that regular exercise would increase immune control of the DRSA infection and, in turn, diminish DRSA-associated responses. We used swimming as the regular exercise because it exerts the same or similar effects as jogging, walking and other aerobic exercises (such as treadmill exercise)^28^. Moreover, it activates the body’s muscles better than treadmill exercise while exerting a smaller weight load on the joints.

DRSA is associated with chronic obstructive pulmonary disease (COPD)^32^, the production of inflammatory cytokines, including tumor necrosis factor α (TNF-α) and interleukin-1β (IL-1β)^33^, as well as the inflammatory marker proteins nitric oxide (NO)^34^ and peroxisome proliferator-activated receptor gamma coactivator 1-α (PGC-1α)^35^. We studied changes in cell morphology, and the expression levels of inflammatory marker proteins, cytokines and cathelicidin AMP in whole blood, adipocytes and whole lungs using ELISA assays, hematoxylin and eosin (H&E) staining, immunohistochemistry (IHC), NO assays and western blot analysis.

## Results

### Levels of TNF-α and IL-1β in whole blood following DRSA infection

**Figure 1:**
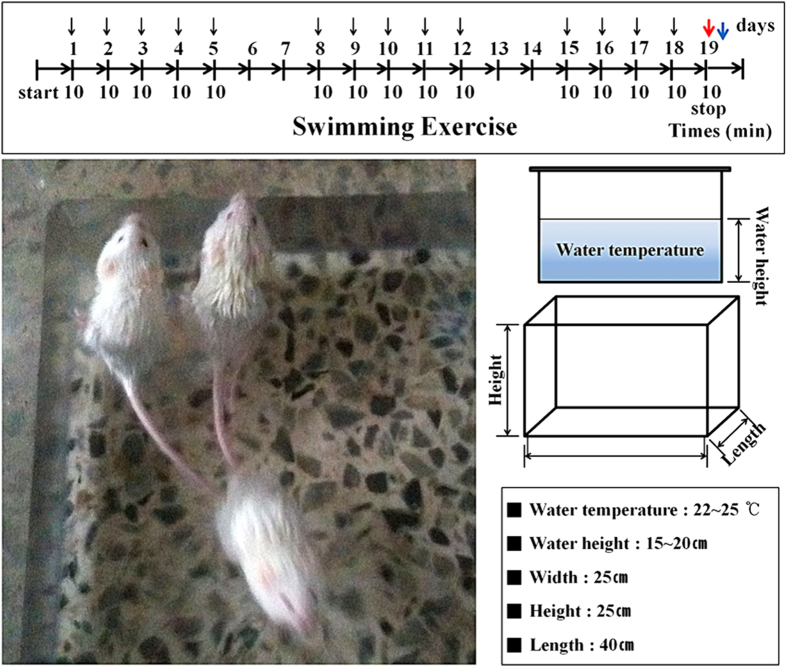
Experimental schedule for swimming and DRSA infection in ICR mice. The mice performed swimming exercise continuously for 10 min/day, 5 days/week for 3 weeks (black arrows). The mice were sacrificed (blue arrow) 6 h after intraperitoneal injection with DRSA (1 × 10^7^ cfu/ml) (red arrow).

**Figure 2:**
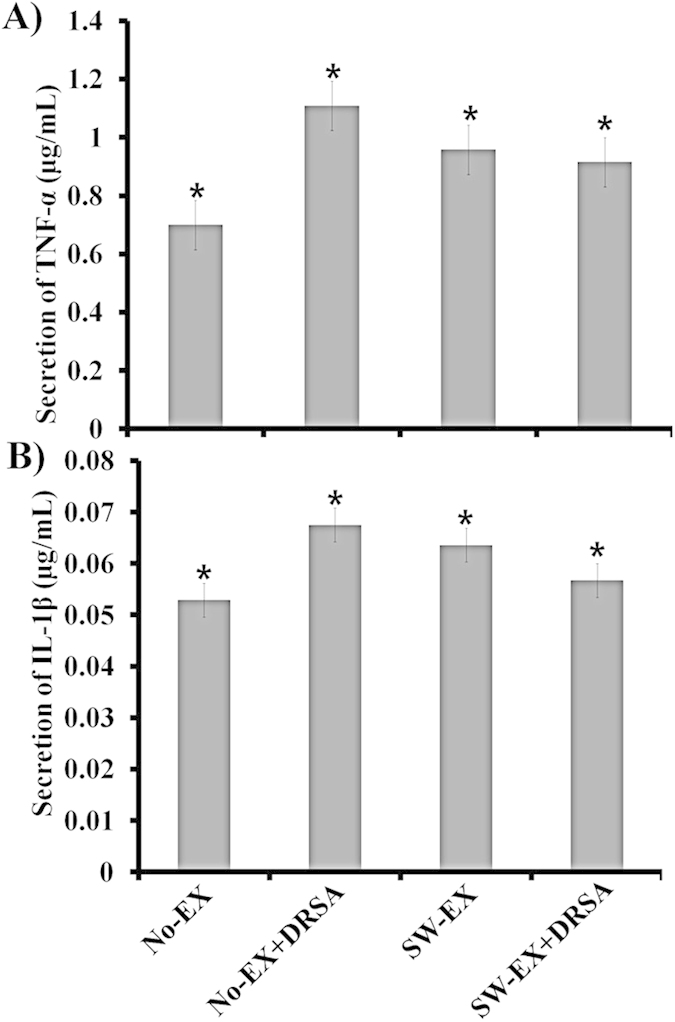
Expression of TNF-α (A) and IL-1β (B) following induced inflammation in ICR mice. Mice were treated with DRSA (1 × 10^**7**^ cfu/ml), after which TNF-α (**A**) and IL-1β (**B**) levels were determined in serum samples from No-EX and SW-EX mice using an ELISA. *P < 0.05 (one-way ANOVA).

The effects of swimming exercise (SW-EX) (10 min/day, 5 days/week for 3 weeks) on induced inflammation were examined using ICR mice infected with DRSA (Fig. 1). Within 6 h after intraperitoneal injection of DRSA (1 × 10^7^ cfu/ml), blood levels of two pro-inflammatory cytokines, TNF-α and IL-1β, were significantly higher in No-EX + DRSA mice than in the No-EX group, but were not significantly elevated in SW-EX or SW-EX + DRSA groups (Fig. 2). Moreover, serum TNF-α and IL-1β levels were lower in SW-EX + DRSA mice than SW-EX mice.

### TNF-α, IL-1β and cathelicidin levels in visceral fat

**Figure 3:**
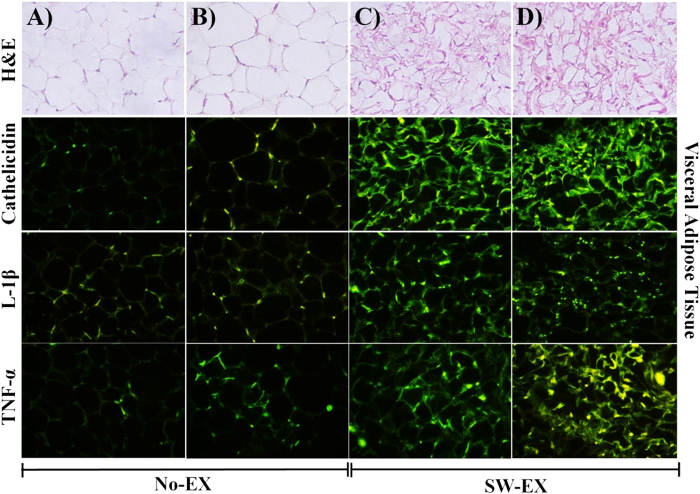
Adipocyte morphology and expression of cathelicidin, IL-1β and TNF-α in visceral adipose tissue following induced inflammation. Visceral fat was extracted from No-EX and SW-EX mice, sectioned in paraffin, stained with H&E and subjected to immunohistochemistry. (**A**) No-EX, (**B**) No-EX + DRSA, (**C**) SW-EX, (**D**) SW-EX + DRSA.

Obesity-induced inflammation within organ tissues serves as the basis for metabolic syndrome^36^,^37^. In addition obesity increases ones susceptibility infection drug-resistant bacteria and the associated pathology^31^,^32^. Figure 3 shows the effect of intraperitoneal injection of DRSA on expression of TNF-α, IL-1β and cathelicidin in the visceral fat of No-EX and SW-EX mice. Immunostaining for TNF-α, IL-1β and cathelicidin shows that within 6 h after injection of DRSA, expression of all three proteins was increased in both No-EX and SW-EX mice. In the SW-EX mice, however, the adipocytes were smaller in size and appeared to express higher levels of TNF-α, IL-1β and cathelicidin than No-EX mice.

### Pulmonary production of nitric oxide (NO)

**Figure 4:**
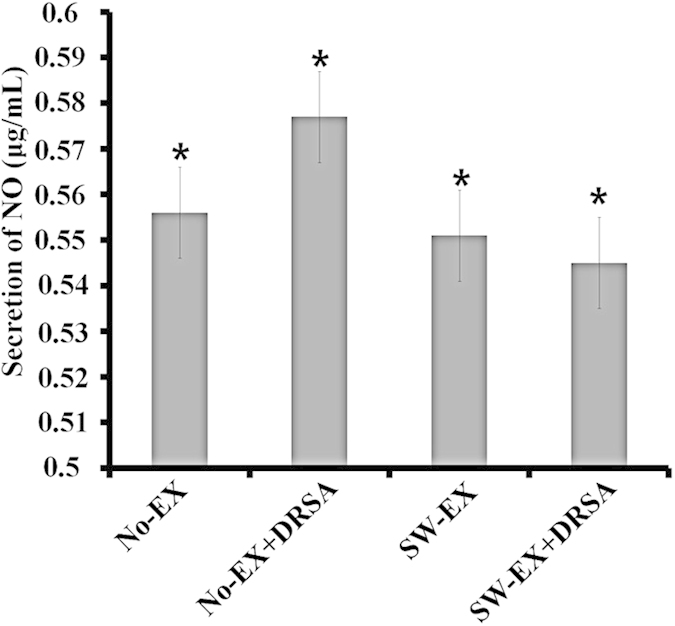
Pulmonary NO production following induced inflammation. Lung tissue extracts were analyzed for NO following induced inflammation in No-EX and SW-EX mice. *P < 0.05 (one-way ANOVA).

To evaluate inflammation of the lungs (pneumonitis) of ICR mice infected with DRSA. We measured NO levels in lung tissue extracts as an index of pneumonitis in No-EX and SW-EX mice (Fig. 4). The results show that intraperitoneal injection of DRSA strongly increased NO production in No-EX mice, but had little or no effect on pulmonary NO production in SW-EX mice. It thus appears that regular exercise may exert a protective effect against pneumonitis induced by DRSA infection in ICR mice.

### Pulmonary expression of pro-inflammatory cytokines and inflammation markers

**Figure 5:**
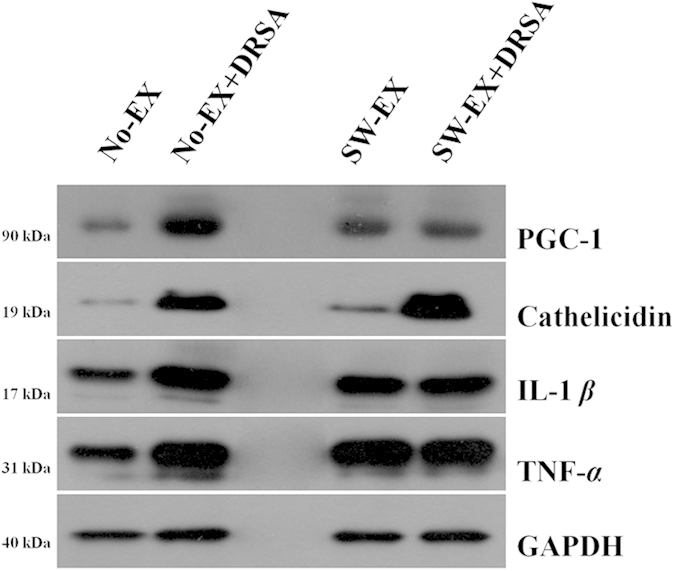
Western blot analysis of pro-inflammatory cytokines, cathelicidin and PGC-1 following induced inflammation. Pulmonary expression of PGC-1, cathelicidin, TNF-α and IL-1β was analyzed by western blotting following induced inflammation in No-EX and SW-EX mice.

Intraperitoneal injection of DRSA led to substantial upregulation of pulmonary TNF-α and IL-1β expression in No-EX mice. By contrast, DRSA infection had little effect on pulmonary expression of TNF-α and IL-1β in SW-EXE mice, although the baseline levels of the two cytokines were already higher than in No-EX mice. Similarly, DRSA infection enhanced pulmonary expression of PGC-1, a protein marker expressed by macrophages and PMNs during inflammatory responses^27^,^28^, in No-EX but not SW-EX mice (Fig. 5). DRSA infection also sharply increased cathelicidin expression in both No-EX and SW-EX mice, as compared with untreated controls.

### Evaluation of morphological changes and expression of cathelicidin in the lungs

**Figure 6:**
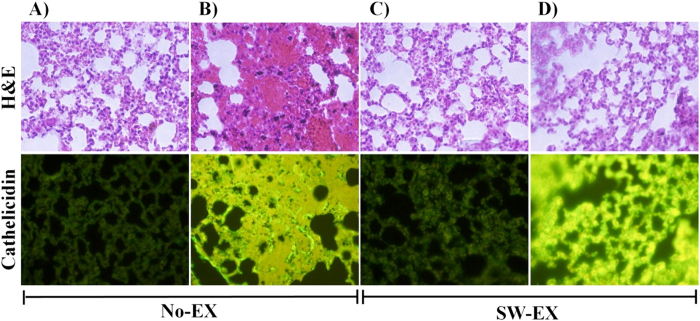
Expression of cathelicidin in lung tissue following induced inflammation. Shown are paraffin-sections of lungs from No-EXE and SW-EX mice collected 6 h after infection with DRSA. The sections were stained with H&E and subjected to immunohistochemistry. (**A**) No-EX, (**B**) No-EXE + DRSA, (**C**) SW-EX, (**D**) SW-EX + DRSA.

Finally, we examined the histological changes in the lungs of mice following intraperitoneal injection of DRSA. We found that in lung tissue from No-EX mice, the induced inflammation led to hemorrhage, infiltration of inflammatory cells (e.g., macrophages), alveolar swelling and production of cathelicidin (Fig. 6A,B). These inflammatory changes were not seen in lung tissue from SW-EX mice, although levels of cathelicidin were strongly upregulated.

## Discussion

It is now recognized that the chronic inflammation associated with obesity is mediated in part by various chemokines, hormones and cytokines (such as TNF-α and IL1-β), and that it plays a key role in a constellation of diseases that includes hypertension, type 2 diabetes and cardiovascular disease, and is often referred to as metabolic syndrome^38^,^39^,^40^,^41^,^42^,^43^. Obesity thus appears to weaken the immune system while inducing a chronic inflammatory response. A key mediator of chronic inflammatory disease is elevated triglyceride (TG) within adipocytes^36^. TG is cleaved by lipases secreted from the pancreas into free fatty acids (FFAs) and glycerol^37^. FFAs induce inflammation by binding to TLR-2 and TLR-4 receptors on immune cells (e.g., macrophages) in blood vessels and stimulating the release of pro-inflammatory cytokines such as TNF-α, IL-1β and IL-6^44^. On the other hand, regular exercise improves immunity^29^ through moderate induction of pro-inflammatory cytokines (e.g., TNF-α, IL-1β and IL-6) in skeletal muscle^45^. Reactive oxidative species (ROS) are also produced^46^ during exercise, which in turn activate c-Jun N-terminal kinases (JNK)^47^. Collectively, these processes are associated with skeletal muscle injury and tissue repair^30^, which induces myofibrillar protein synthesis and muscle hypertrophy through activation of mammalian target of rapamycin complex 1 (mTORC1)^48^, and promotes organ function^49^,^50^. Regular exercise also appears to shrink adipocytes and reduce obesity by inducing β-oxidation^51^.

Infectious microorganisms also elicit acute inflammatory responses mediated by cytokines secreted from immune cells, akin to inflammation associated with obesity^52^. In this study, we examined the effect of 3 weeks of exercise (swimming) on the immune response induced by infection with DRSA. Our findings show that regular exercise suppresses infection-induced inflammation, as indicated by reductions in the levels in two inflammatory cytokines (TNF-α and IL1-β) and two inflammation markers (NO and PGC-1), and it increases cathelicidin levels in the blood, adipose tissue and whole lung tissue. Earlier reports indicate that production of TNF-α and IL1-β is significantly increased by regular exercise^53^,^54^,^55^, resulting in improved immune responses, prevention of various diseases, increased signal transduction and reduced body mass through β-oxidation^56^,^57^. Secretion of large amounts of cytokines stimulated by infectious pathogens results in strong activation of immune cells^58^. TNF-α and IL-1β, in particular, are key mediators of the signal transduction initiating immediate immune cell activity upon release from the site of infection^59^,^60^. We observed that serum TNF-α and IL-1β levels were lower in SW-EX and SW-EX + DRAS mice than No-EX + DRSA mice, and were even lower in SW-EX + DRSA than SW-EX mice. We also observed that production of TNF-α and IL-1β was reduced in exercising mice, while cathelicidin production was increased, suggesting that suppression of the infection by cathelicidin led to a decrease in cytokine production. We therefore suggest that the immediate action (autocrine and/or paracrine) of cathelicidin on the infected tissues contributed to a reduction in serum cytokine levels.

On the other hand, SW-EX mice exhibited higher levels of TNF-α and IL-1β in adipose tissue. This is thought to reflect increased proliferation and/or recruitment of macrophages (including a switch from the M2 to the M1 phenotype) to the area around adipocytes as a result of β-oxidation of free fatty acids accomplished through regular exercise^58^,^60^. Regular exercise would also be expected to increase expression of cathelicidin by infiltrating macrophages in adipose tissue. Consistent with that idea, we observed significantly greater cathelicidin production in both uninfected and infected SW-EX mice than in No-EX mice. Furthermore, the cathelicidin production would also be expected to influence whole-body immune responses via the lymphatic system and blood vessels following infection with DRSA. This is perhaps reflected by pulmonary NO production, which is known to be enhanced by inflammation caused by infectious disease and/or obesity^38^. When we measured pulmonary NO following DRSA infection of No-EX and SW-EX mice, we observed that although NO levels did not differ between the two groups under basal conditions, they were significantly higher in No-EX mice infected with DRSA suggesting lack of exercise may increase the likelihood of adverse whole-body immune responses that can underlie septic shock.

It was previously reported that pulmonary levels of TNF-α, IL-1β and the inflammation marker PGC-1 are all increased following infection with microorganisms such as *S. aureus*, often leading to sepsis and death^61^,^62^,^63^,^64^,^65^. However, cathelicidin produced by macrophages and PMNs, along with other AMPs such as defensin and histatin, exert strong antimicrobial effects against microorganisms such as *S. aureus* and *Pseudomonas aeruginosa*^27^,^54^. When we compared the production of TNF-α and IL1-β, PGC-1 and cathelicidin in whole lung tissue from No-EX and SW-EX ICR mice following DRSA infection, we found that DRSA infection had little effect on pulmonary expression of TNF-α and IL-1β in SW-EXE mice, though their baseline levels were already higher than in No-EX mice. Likewise, DRSA infection had little effect on pulmonary expression of PGC-1. By contrast, cathelicidin levels were increased to a substantially greater (more than 3-fold) degree in SW-EX than No-EX mice following DRSA infection. These findings suggest that regular exercise increases production of cathelicidin, thereby enhancing the endogenous antimicrobial response against bacterial infection and, in turn, suppressing production of inflammatory cytokines and inflammation markers.

We propose that regular exercise increases cathelicidin production by immune cells in adipose and lung tissues, which also produce TNF-α and IL1-β. However, the increased cathelicidin prevents the inflammation-induced secretion of TNF-α, IL1-β, PGC-1 from immune cells by disrupting bacterial membranes and binding to lipoteichoic acid (LTA) on the bacterial cell surface. In addition, it was previously reported that activation of skeletal muscle during regular exercise leads to increases in the serum levels of various cytokines^66^, which in turn mediate other inflammatory responses^32^,^33^,^67^. By contrast, we found that irrespective of DRSA infection-induced inflammation, serum cytokine levels in ICR mice subjected to regular exercise (SW-EX + DRSA and SW-EX) were lower than in infected, unexercised control mice (No-EX + DRSA). In fact, serum TNF-α and IL-1β levels in the SW-EX + DRSA and SW-EX groups did not significantly differ from those in the uninfected and unexercised control group (No-EX mouse). Notably, TNF-α and IL-1β levels appeared to be even lower in SW-EX + DRSA than SW-EX mice (Fig. 2). We think that the lower serum levels of pro-inflammatory cytokines reflects the immediate stimulus and engagement (autocrine and/or paracrine) of the immune cell receptors (such as macrophages) by the cytokines in the infected animals, and that the skeletal muscle damage caused by the exercise enhanced that effect^68^. Ultimately, the effective immune cell response to the infectious microorganism (DRSA), including release of cathelicidin, led to rapid mitigation of cytokine release and lower serum levels than in the uninfected control.

**Figure 7:**
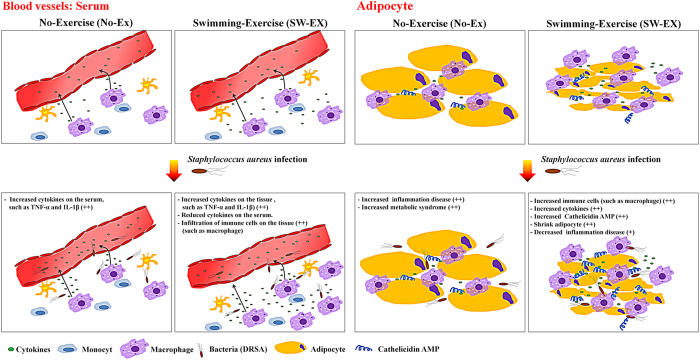
Schematic of the proposed effects of regular exercise on induced inflammation.

β-oxidation of free fatty acids not only leads to shrinkage of adipocytes during regular exercise, but reduces inflammation, which has a preventive effect on diseases such as diabetes, cardiovascular disease and stroke, among others^60^,^69^ (Fig. 7), and it also improves immune system defenses and decreases susceptibility to infection.

## Methods

### Experimental design and swimming exercise training

Male ICR mice were used for this study. The ambient air temperature was maintained at 22–25 °C with 40–60% humidity and a 12 h dark-light cycle. The swimming exercise procedure conformed to the “Resource Book for the Design of Animal Exercise Protocols” and was approved by the American Physiological Society Committee to Develop an APS Resource Book for the Design of Animal Exercise Protocols. When handling mice, we followed the established guidelines.

Eight-week-old ICR mice were assigned to a no-exercise (No-EX, n = 7) or swim-trained (SW-EX, n = 7) group. The exercise training protocol for the swimming group was as follows: prior to the initiation of training, the mice were allowed access to the swimming pool for 3 min/day for 5 days, which enabled them to become familiar with the environment. After finishing each exercise session, the mice were completely dried to prevent hypothermia. The rectangular tank was 15–25 cm deep, 40 cm long and 25 cm wide. During training, mice in the SW-EX group swam for 10 min/day, 5day/week for 3 weeks. We monitored the mice during the swim to prevent them from climbing, diving, bobbing or floating. Mice in the No-EX group were treated the same in all other regards, but did not undergo swim training^70^,^71^,^72^.

To elicit changes in cytokine, cathelicidin and inflammatory protein expression, inflammation was induced by infecting mice through intraperitoneal injection of DRSA (1 × 10^**7**^ cfu/ml)^19^,^25^,^26^. Negative control mice (No-EX and SW-EX) were administered PBS buffer without bacteria. After 6 h, experimental mice were anesthetized using 0.5–2% isoflurane (the procedure conformed to the “Korean College of Laboratory Animal Medicine”, KCOLAM), and whole blood samples were collected from the heart, and lung and adipose tissue samples were collected through abdominal incisions.

### Measurement of serum TNF-α and IL-1β in ICR mice

TNF-α and IL-1β expression was measured in whole blood from No-EX and SW-EX mice following induction of inflammation with DRSA. Whole blood samples were collected 6 h after the mice were injected with bacteria or vehicle (control). The samples were then centrifuged at 2000 *g* for 10 min at 4 °C to remove the red blood cells and the serum was retained. Levels of TNF-α and IL-1β were measured using specific ELISA kits (Koma Biotech, Seoul, Korea). Absorbance was measured at 450 nm using a microplate reader^63^.

### Immunohistochemistry (IHC) and hematoxylin & eosin staining

Lungs and visceral fat were extracted from mice, washed once with phosphate buffered saline (PBS) and fixed in 4% paraformaldehyde for 24 h at 4 °C. The fixed tissue was then dehydrated through a 50–100% ethanol series (2 h at each step) and 3 incubations (1 h each) in xylene, embedded in paraffin and cut into 4-μm-thick sections using a microtome. Sections were incubated for 30 min at room temperature with primary antibody in 5% bovine serum albumin (BSA). The primary antibodies used were polyclonal mouse anti-cathelicidin (ABfrontier, AB93357), monoclonal mouse anti-TNF-α (ABfrontier, AB1793) and polyclonal mouse anti-IL-1β (ABfrontier, AB1413). The samples were washed with tris-buffered saline containing tween (TBST buffer), incubated with secondary antibody (goat anti-mouse IgG (HRP) LF-SA5001-conjugated), and stained with hematoxylin and eosin (H&E). Stained sections were examined under a fluorescence microscope (Ix71, Olympus, Tokyo, Japan)^64^.

### Measurement of nitric oxide (NO) in lungs

After injecting mice with DRSA or vehicle, samples of whole lung tissues were collected from the No-EX and SW-EX groups and homogenized in one volume of PRO-PREP^TM^ protein extraction solution (iNtRON Biotechnology, Seoul, Korea). After homogenization and centrifugation at 12,000 *g* for 10 min to remove floating contaminants, the supernatant was retained for NO assays. To determine NO levels, Griess reagent was added to the samples, which were then mixed with equal volumes of sulfanilic acid (1% in phosphoric acid) and N-(1-naphthyl) ethylenediamine dihydrochloride (0.1% in DW). Aliquots of the resultant mixture (50 μl) were incubated for 30 min at room temperature with mixing, after which absorbance was measured at 548 nm using a microplate reader. NO levels were calculated against a standard curve constructed using sodium nitrite^73^.

### Western blot analysis

For western blot analysis, proteins collected from whole lung tissue were separated by SDS-PAGE in a 15% polyacrylamide gel for 3 h. The separated proteins were then transferred to PVDF membranes (Bio-Rad, USA) for 1 h at 90 volts. After blocking the membranes overnight at 4 °C with 5% skim milk, they were probed using anti-GAPDH (Santa Cruz Biotechnology, LF-PA0018), polyclonal mouse anti-NF-κB (Santa Cruz Biotechnology, SC-71675), monoclonal mouse anti-TNF-α (ABfrontier, AB1793), polyclonal mouse anti-IL-1β (ABfrontier, AB1413), polyclonal mouse anti-Cathelicidin (ABfrontier, AB93357) and polyclonal mouse anti-PGC-1 (Millipore AB3242). The membranes were then washed with TBST buffer and incubated with secondary antibodies (goat anti-mouse IgG (HRP) LF-SA5001-conjugated). Finally, the blots were developed using a western blot detection kit (LF-QC0103, Abfrontier)^74^.

### Statistical analysis

The data were analyzed using SPSS version 20.0 (Chicago, IL, USA). Values are presented as means ± SD. One-way ANOVA was used to compare TNF-α, IL-1β and NO levels between the No-EX and SW-EX groups. P values < 0.05 were considered significant.

## Additional Information

**How to cite this article**: Lee, J.-K. *et al.* Effect of Regular Exercise on Inflammation Induced by Drug-resistant *Staphylococcus aureus* 3089 in ICR mice. *Sci. Rep.* **5**, 16364; doi: 10.1038/srep16364 (2015).
